# Wnt-11 promotes neuroendocrine-like differentiation, survival and migration of prostate cancer cells

**DOI:** 10.1186/1476-4598-9-55

**Published:** 2010-03-10

**Authors:** Pinar Uysal-Onganer, Yoshiaki Kawano, Mercedes Caro, Marjorie M Walker, Soraya Diez, R Siobhan Darrington, Jonathan Waxman, Robert M Kypta

**Affiliations:** 1 Department of Oncology, Imperial College London, London, UK; 2 Department of Histopathology, Imperial College London, London, UK; 3 Centre for Cooperative Research in Biosciences (CIC bioGUNE), Derio, Spain

## Abstract

**Background:**

Wnt-11 is a secreted protein that modulates cell growth, differentiation and morphogenesis during development. We previously reported that Wnt-11 expression is elevated in hormone-independent prostate cancer and that the progression of prostate cancer from androgen-dependent to androgen-independent proliferation correlates with a loss of mutual inhibition between Wnt-11- and androgen receptor-dependent signals. However, the prevalence of increased expression of Wnt-11 in patient tumours and the functions of Wnt-11 in prostate cancer cells were not known.

**Results:**

Wnt-11 protein levels in prostate tumours were determined by immunohistochemical analysis of prostate tumour tissue arrays. Wnt-11 protein was elevated in 77/117 of tumours when compared with 27 benign prostatic hypertrophy specimens and was present in 4/4 bone metastases. In addition, there was a positive correlation between Wnt-11 expression and PSA levels above 10 ng/ml. Androgen-depleted LNCaP prostate cancer cells form neurites and express genes associated with neuroendocrine-like differentiation (NED), a feature of prostate tumours that have a poor prognosis. Since androgen-depletion increases expression of Wnt-11, we examined the role of Wnt-11 in NED. Ectopic expression of Wnt-11 induced expression of NSE and ASCL1, which are markers of NED, and this was prevented by inhibitors of cyclic AMP-dependent protein kinase, consistent with the known role of this kinase in NED. In contrast, Wnt-11 did not induce NSE expression in RWPE-1 cells, which are derived from benign prostate, suggesting that the role of Wnt-11 in NED is specific to prostate cancer. In addition, silencing of Wnt-11 expression in androgen-depleted LNCaP cells prevented NED and resulted in apoptosis. Silencing of Wnt-11 gene expression in androgen-independent PC3 cells also reduced expression of NSE and increased apoptosis. Finally, silencing of Wnt-11 reduced PC3 cell migration and ectopic expression of Wnt-11 promoted LNCaP cell invasion.

**Conclusions:**

These observations suggest that the increased level of Wnt-11 found in prostate cancer contributes to tumour progression by promoting NED, tumour cell survival and cell migration/invasion, and may provide an opportunity for novel therapy in prostate cancer.

## Background

Wnt genes code for secreted signalling proteins that are responsible for the development and repair of many organs in the body and aberrant activation of Wnt signalling is implicated in tumorigenesis [1]. We have previously reported increased expression of Wnt-11 mRNA in androgen-independent prostate cancer (PCa) [2]. Wnt-11 is best known for its role during development, for example, it is required for convergent extension movements during gastrulation [3] and kidney morphogenesis [4]. In addition, cell-based assays have demonstrated that Wnt-11 promotes cardiac differentiation [5], increases proliferation, migration and transformation of intestinal epithelial cells [6], reduces apoptosis in breast cancer cells [7] and increases cell viability in chinese hamster ovary (CHO) cells [8]. The signals downstream of Wnt-11 are not fully characterised. Wnt-11 has been reported to inhibit JNK and NF-kappaB [8], activate PKC and JNK [9] and activate cAMP response element binding protein (CREB) family members [10]. Wnt-11 does not appear to stabilise β-catenin and is frequently found to inhibit 'canonical' Wnt/β-catenin signalling [2,8,11].

Despite its high level of expression in androgen-independent PCa cells, ectopic expression of Wnt-11 inhibits the growth of the androgen-dependent LNCaP cells [2]. The morphology of Wnt-11-transfected LNCaP cells resembles that of androgen-depleted LNCaP cells as they undergo neuroendocrine-like differentiation (NED) [12], which we previously reported induces expression of endogenous Wnt-11 [2]. This raised the possibility that Wnt-11 may promote NED. Neuroendocrine (NE) cells constitute a minor cell population in the normal prostate that is thought to regulate prostatic growth and differentiation. However, in prostate tumours, the number of NE-like cells correlates with tumour progression, poor prognosis and the androgen-independent state [12,13]. These and other observations have led to the suggestion that PCa cells transdifferentiate to become NE-like. Agents that induce NED elevate intracellular levels of cyclic AMP (cAMP), and it has been postulated that cAMP-mediated signalling is a primary pathway of NED *in vivo *[14]. Here, we have further investigated the function of Wnt-11 and show that it promotes NED in a PKA-dependent manner and promotes prostate cancer cell survival, migration and invasion.

## Results

### Increased expression of Wnt-11 in prostate tumours

**Figure 1:**
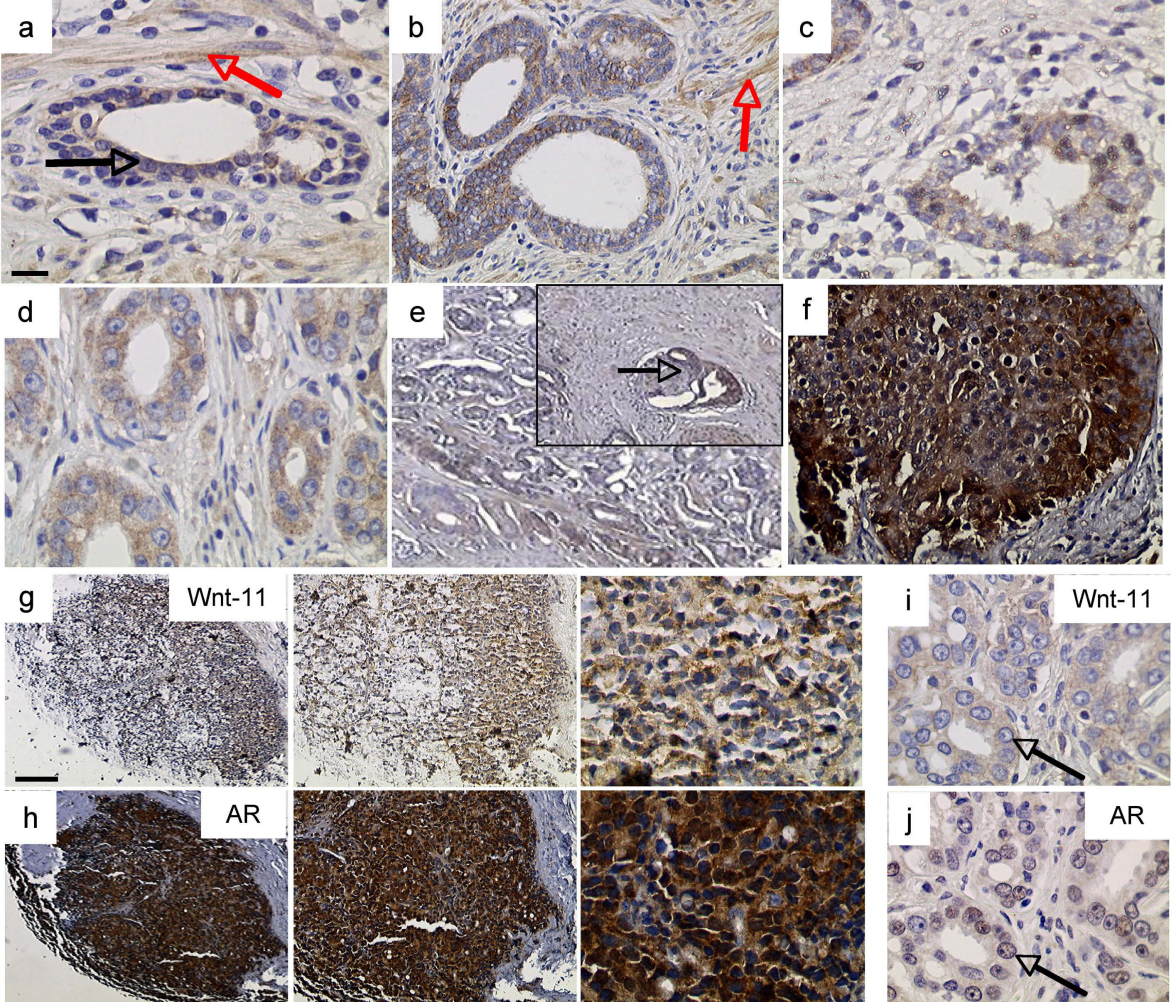
**Wnt-11 protein expression in prostate and prostate cancer**. Immunohistochemical analysis of Wnt-11 in (a) benign prostate; faint cytoplasmic expression (black arrow) and stromal smooth muscle (red arrow) are indicated, (b-d) examples of Gleason 4 tumours, (e) Gleason 4 tumour with perineural invasion (inset arrowed), (f) a strongly positive Gleason 5 tumour, (g, h) adjacent sections of a prostate cancer bone metastasis showing Wnt-11 (g) and androgen receptor (AR, h), and (i, j) adjacent sections of a Gleason 4 tumour showing Wnt-11 (i) and AR (j); arrows show PCa cell co-expressing Wnt-11 and nuclear AR. Scale bar (a-f, i, j): 50 μm, (g, h, left to right): 200 μm, 75 μm and 25 μm.

In order to determine whether Wnt-11 protein levels are elevated in patient tumours, we used anti-Wnt-11 antibodies to localise Wnt-11 expression in sections taken from human prostate and prostate tumour tissue (Figure 1). Benign prostate sections (27 cases) were either negative (not shown) or exhibited weak expression of Wnt-11 in luminal epithelial cells and in some smooth muscle cells (Figure 1a). Malignant prostate showed stronger expression of Wnt-11 in luminal epithelial cells (Figures 1b, c). Immunohistochemical analysis of Wnt-11 in tumour tissue arrays indicated that the level of Wnt-11 was elevated in 77/117 (66%) of tumours (Figures 1b - 1d), with particularly strong staining in 28/117 (24%) of cases (Figure 1f). In addition, Wnt-11 was detected in 2/2 examples of perineural invasion (Figure 1e) and in 4/4 bone metastases (Figure 1g, high expression of the androgen receptor (AR) is shown in an adjacent section) suggesting a possible role for Wnt-11 in invasion and/or metastasis. Finally, Wnt-11 and AR were found to be co-expressed in prostate tumour cells (Figures 1i, j). The level of Wnt-11 expression was generally higher in PCa compared to benign prostate. Analysis in relation to conventional prognostic indices of PCa showed a negative correlation with Gleason grade; Wnt-11 was more frequently found in Gleason 3 tumours than in Gleason 4 tumours and in Gleason < 8 compared to Gleason > 8, suggesting that Wnt-11 is not a general marker for tumour dedifferentiation. However, Wnt-11 was significantly elevated in tumours from patients with PSA levels above 10 ng/ml (Table 1).

**Table 1:** Comparison of Wnt-11 protein expression and tumour stage

Comparison (Wnt-11 --ve versus Wnt-11 +ve)	*p *value (Chi-squared test)
PSA < 10 ng/ml versus PSA > 10 ng/ml	0.0001
Gleason 4 versus Gleason 3	0.005
Gleason 5 versus Gleason 4	0.3
Gleason sum 7 versus Gleason sum 6	0.01
Gleason sum >8 versus Gleason sum <8	0.02
PNI +ve versus PNI -ve	0.6

Tumour samples from 117 patients were analysed. 77/117 (66%) of tumours contained Wnt-11-positive epithelial cells, 28/117 (24%) of tumours were strongly Wnt-11 positive and 4/4 bone metastases were Wnt-11 positive. Wnt-11 expression correlated with increased serum PSA but lower Gleason score. Statistical significance was determined using chi-squared test and results were considered significant for p < 0.05. PSA, prostate-specific antigen; PNI, perineural invasion.

### Wnt-11 promotes neuroendocrine-like differentiation in prostate cancer cells

**Figure 2:**
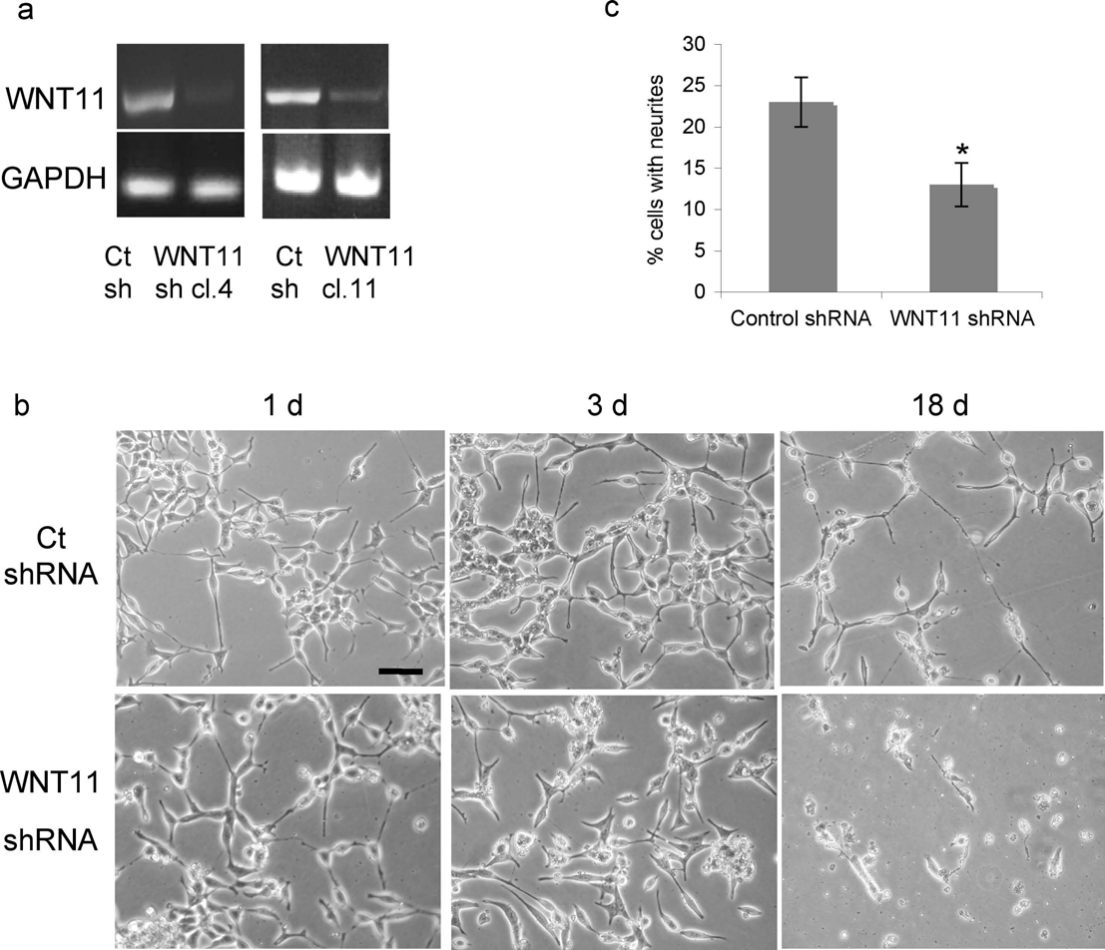
**Silencing of Wnt-11 reduces survival of androgen-depleted LNCP cells**. (a) RT-PCR of *WNT11 *and *GAPDH *in LNCaP cell lines stably expressing control (Ct) shRNA or WNT11 shRNA (clones 4 and 11) cultured for 7 days in hormone-depleted medium. (b) Images of control shRNA or WNT11 shRNA (clone 11) LNCaP cells cultured for the indicated times in hormone-depleted medium. Scale bar 200 μm. Similar results to those shown were obtained using three independent WNT11 shRNA clones. (c) Quantitation of neurite outgrowth of control shRNA or WNT11 shRNA (clone 11) LNCaP cells cultured for 4 days in hormone-depleted medium. The data presented are the means (+/- SD) derived from counting three fields of 100 cells; * *p *< 0.05.

We noted that the morphology of Wnt-11-transfected LNCaP cells resembled that of androgen-depleted LNCaP cells as they undergo NED, raising the possibility that Wnt-11 may be involved in NED. To test this possibility, we examined the effects of downregulation of endogenous Wnt-11 in LNCaP cells. LNCaP cell lines were generated expressing WNT11 shRNA or a non-silencing control shRNA. RT-PCR analysis confirmed upregulation of Wnt-11 upon hormone-depletion in control shRNA cells but not in WNT11 shRNA cells (Figure 2a). Culture of control shRNA cells in hormone-depleted medium for a longer period of time resulted in growth arrest and generation of neurite-like processes (Figure 2b), as previously described for parental LNCaP cells [15,16]. In contrast, hormone-depletion of WNT11 shRNA cells produced an initial morphological response after one day in hormone-depleted medium (Figure 2b), but thereafter the number of neuritic processes decreased, with a significant reduction in the percentage of cells with neurites after 4 days (Figure 2c). After 18 days in hormone-depleted medium, control shRNA cells remained viable and had long neurites, whereas almost all the WNT11 shRNA cells had died (Figure 2b).

**Figure 3:**
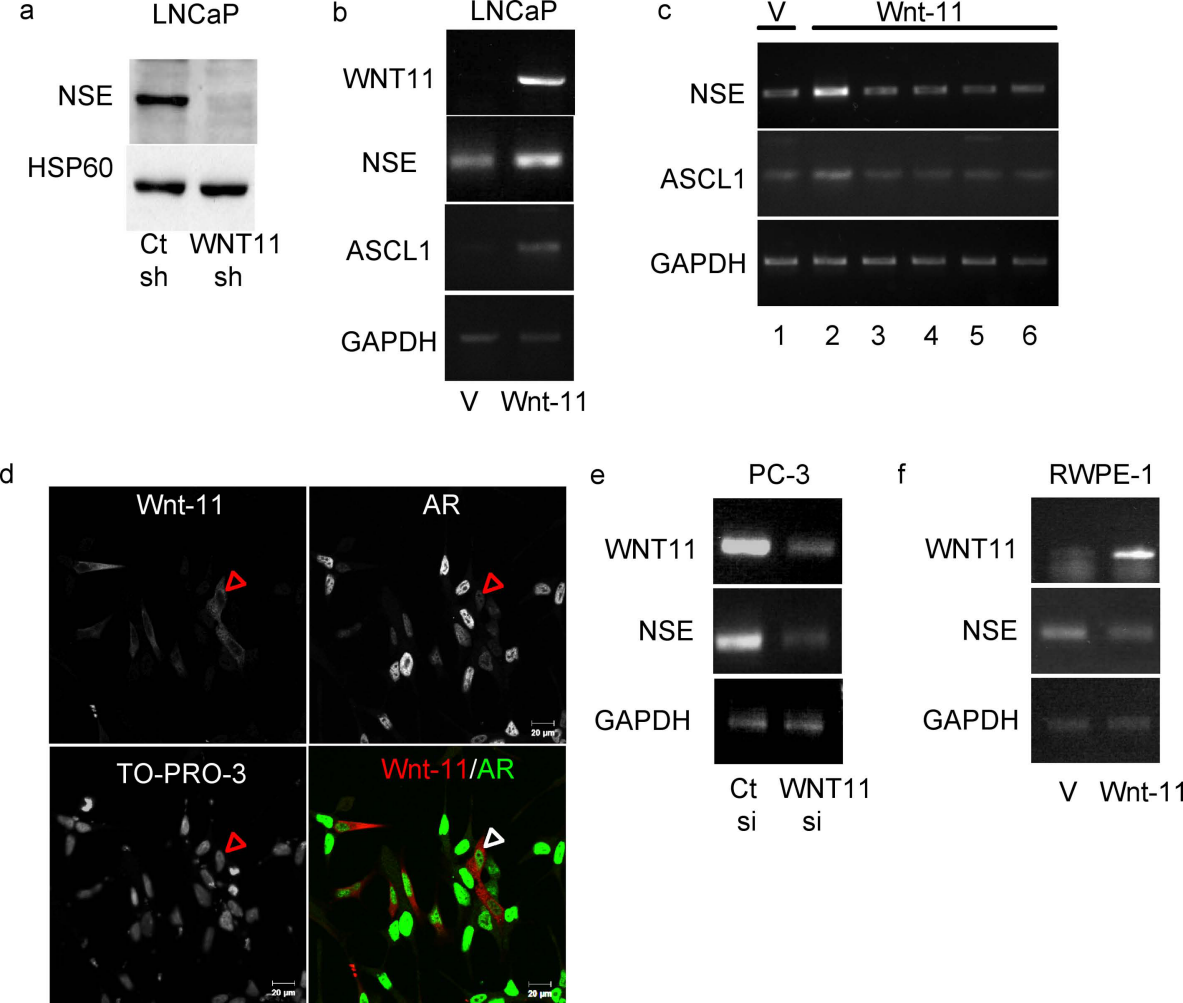
**Wnt-11 promotes neuroendocrine-like differentiation in PCa cells**. (a) Extracts from control shRNA and WNT11 shRNA LNCaP cells cultured for 3 days in hormone-depleted medium were probed by western blotting for neuron-specific enolase (NSE). The blot was then stripped and re-probed for HSP60. (b) RT-PCR for *WNT11*, *NSE*, *ASCL1 *and *GAPDH *expression after transfection of LNCaP cells either with empty vector (V) or Wnt-11 expression plasmid. (c) RT-PCR for *NSE*, *ASCL1 *and *GAPDH *expression levels in LNCaP cells transfected either with empty vector (V, lane 1) or Wnt-11 expression plasmid (lanes 2-6) and cultured in the presence of carrier (DMSO, lanes 1 and 2), H89 (lane 3), KT5720 (lane 4) Rp-cAMPs (lane 5) or PKI (lane 6). (d) Immunostaining for Wnt-11 (red) and AR (green) in LNCaP cells transfected with Wnt-11 expression plasmid. Red arrows indicate a cell with high Wnt-11 and low AR. TO-PRO-3 was used to stain nuclei. (e) RT-PCR for *WNT11*, *NSE *and *GAPDH *in PC3 cells transfected with control siRNA (Ct si) or WNT11 siRNA (WNT11 si). (e) RT-PCR for *WNT11*, *NSE *and *GAPDH *in RWPE-1 cells transfected as in (b) with empty vector (V) or Wnt-11 expression plasmid.

To determine if the differences in neurite outgrowth resulted from an inability of WNT11 shRNA cells to undergo NED, extracts from cells cultured in hormone-depleted medium were probed for a marker of NED, neuron-specific enolase (NSE) (Figure 3a). Control shRNA cells cultured in hormone-depleted medium expressed NSE, whereas hormone-depleted WNT11 shRNA expressed very little NSE. Thus, endogenous Wnt-11 appears to be required for expression of NSE, a marker for NED, in hormone-depleted LNCaP cells.

Ectopic expression of Wnt-11 reduces proliferation of LNCaP cells [2] and cell lines overexpressing Wnt-11 could be maintained for only a limited number of passages, suggesting that Wnt-11 induces LNCaP cell growth arrest or differentiation. To determine whether increased expression of Wnt-11 is sufficient to promote NED, LNCaP cells were transfected with a Wnt-11 expression vector and the levels of expression of *NSE *and a second gene, *ASCL1*, whose expression is increased during NED [17] were measured (Figure 3b and Additional File 1). Wnt-11 increased expression of *NSE *and *ASCL1*, suggesting that Wnt-11 is sufficient to induce NED in LNCaP cells. Androgen depletion activates NED in LNCaP cells by increasing cellular levels of cAMP, leading to activation of PKA [15]. Therefore we tested the effect of inhibiting cAMP signalling on Wnt-11 induction of these same genes. Wnt-11 induction of *NES *and *ASCL1 *was reduced to baseline levels upon treatment with four independent PKA inhibitors (H89, KT5720, Rp-cAMPS and PKI, Figure 3c), suggesting that Wnt-11 induction of NED requires activation of PKA.

NE-like differentiation is associated with a loss of AR expression in both LNCaP cells and human tumours [13]. Since Wnt-11 inhibits AR activity, we determined whether ectopic expression of Wnt-11 in LNCaP cells affected the expression level or localisation of AR. The level of AR expression was reduced in LNCaP cells expressing Wnt-11, but its subcellular localisation was not affected (Figure 3d). This suggests that Wnt-11 induction of NE-like differentiation in LNCaP cells might result from its ability to reduce AR expression. However, LNCaP cells expressing Wnt-11 still expressed some AR. In order to test whether Wnt-11 might also play a role in NED in AR-negative PCa cells, we conducted experiments using PC3 cells, an AR-negative, PCa cell line that expresses high levels of Wnt-11 [2] and displays some characteristics of NE cells [13]. It was not possible to generate PC3 cell lines stably expressing shRNAs and the transfection efficiency of the shRNA vectors was too low to achieve silencing of WNT11. Therefore, WNT11 was silenced by transient transfection of siRNA oligonucleotides. Silencing of WNT11 in PC3 cells resulted in a reduction of *NSE *mRNA levels 24 h after transfection (Figure 3e). These results suggest that Wnt-11 plays a role in NED of both AR-positive and AR-negative prostate cancer cells. In order to determine if Wnt-11 was also involved in NED in untransformed prostate epithelial cells, we expressed Wnt-11 in RWPE-1 cells, which are derived from normal prostate and exhibit features of prostate progenitor cells, including the ability to differentiate into NE cells [18]. Expression of Wnt-11 did not affect the level of *NSE *mRNA in RWPE-1 cells (Figure 3f). This difference did not result from differences in the amount of Wnt-11 protein expressed, since transfection efficiency was similar in LNCaP and RWPE-1 cells (Additional File 2). This suggests that Wnt-11 promotes NED in PCa cells but not in untransformed prostate epithelial cells.

### Silencing of WNT11 increases apoptosis in prostate cancer cells

**Figure 4:**
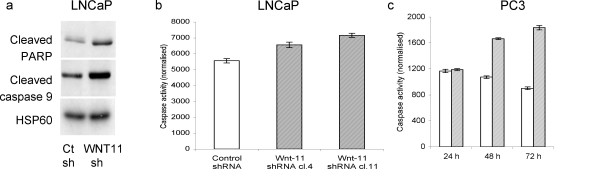
**Downregulation of Wnt-11 leads to apoptosis in prostate cancer cells**. (a) Extracts from control shRNA and WNT11 shRNA LNCaP cells grown in androgen-depleted medium for 7 days were probed by western blotting for cleaved PARP, cleaved caspase-9 and HSP60. (b) Caspase assays from control shRNA and WNT11 shRNA LNCaP cells grown in androgen-depleted medium for 4 days. Caspase activity was significantly higher in WNT11 shRNA cells (shaded bars) compared to in control shRNA cells (n = 6; p < 0.05). (c) Caspase assays from PC3 cells transfected with control siRNA (white bars) or WNT11 siRNA (shaded bars). Caspase activity was significantly increased in WNT11 siRNA transfected cells at 2 days and 3 days (n = 4; p < 0.001). All data are shown as means ± standard deviation.

Silencing of WNT11 appeared to reduce the viability of LNCaP cells grown in androgen-depleted medium (Figure 2b). In order to determine if this reflected an effect on apoptosis, cell extracts from androgen-depleted control and WNT11 shRNA cells were probed for cleaved PARP and cleaved caspase 9, which are frequently used as indicators of apoptosis. The levels of both cleaved proteins were elevated in androgen-depleted WNT11 shRNA cells when compared to controls (Figure 4a). Furthermore, caspase assays conducted using extracts from WNT11 shRNA cells and control shRNA cells grown in androgen-depleted medium confirmed that loss of Wnt-11 increases apoptosis in androgen-depleted cells (Figure 4b). To determine whether endogenous Wnt-11 also protected PC3 cells from apoptosis, PC3 cells were transfected with control or WNT11 siRNA oligonucleotides and then assayed for caspase activity. Caspase activity was higher in WNT11 siRNA cells compared to in control cells (Figure 4c), indicating that endogenous Wnt-11 also plays a role in preventing apoptosis in this androgen-independent PCa cell line.

### Wnt-11 promotes prostate cancer cell migration and invasion

**Figure 5:**
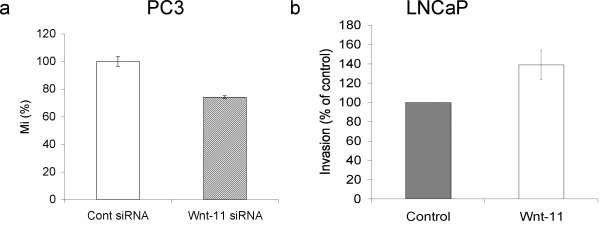
**Wnt-11 promotes prostate cancer cell migration and invasion**. (a) PC3 cells were transfected for 72 hours with control or WNT11 siRNAs, plated on Transwell filters and the extent of migration determined. WNT11 siRNA decreased transverse migration (p = 0.02; n = 9). Results are plotted as Migration Index (Mi), which is the percentage of migrated cells compared to the total number of cells seeded. (b) LNCaP cells were transfected with empty vector or Wnt-11 expression plasmid, plated on Matrigel-coated transwell filters and the extent of invasion determined. The extent of invasion was normalised to that in control cells (p = 0.01; n = 5). The total cell number did not change during the course of the experiment.

Wnt-11 has been reported to affect migration during development and in some cell lines [6,19,20]. To determine if Wnt-11 was involved in PC-3 cell migration, cells were transfected with control or WNT11 siRNA oligonucleotides and assayed for their ability to migrate through Transwell filters (Figure 5a). Compared to the control siRNA, WNT11 siRNA reduced PC3 cell transverse migration, suggesting that endogenous Wnt-11 also plays a role in migration of this metastatic PCa cell line. LNCaP cells, in contrast to PC3 cells, do not migrate and are not normally invasive. However, LNCaP cells can be induced to invade by secreted factors, for example, neurotensin and components of FCS [21]. In order to determine if Wnt-11 overexpression promotes invasion, LNCaP cells were transfected with vector or Wnt-11 expression plasmids and, after two days to allow for Wnt-11 expression, plated on Matrigel-coated filters in the presence of 1% FCS and invasion assays performed. Expression of Wnt-11 increased the number of invading LNCaP cells compared to control cells (Figure 5b). MTT assays indicated that there were no differences in the total numbers of cells during the assay (data not shown). Together, these results indicate that Wnt-11 not only plays roles in NED and survival of prostate cancer cells, but also in their ability to migrate and invade.

## Discussion

Our results suggest that Wnt-11 can endow prostate tumour cells with three properties important for PCa progression, namely, increased resistance to apoptosis, neuroendocrine-like differentiation and increased motility. The increased resistance to apoptosis provides a survival mechanism for PCa cells not only under conditions of hormone-depletion but also in castrate-resistant PCa. Wnt-11 has previously been shown to be important for the survival and/or viability of MCF-7 breast cancer cells [7] and CHO cells [8], suggesting that this is a general role for Wnt-11. It is not clear whether the survival function of Wnt-11 in PCa is direct or results from its effects on NED. WNT11 siRNA reduced NSE expression in PC3 cells after 24 h, before it significantly increased caspase activity (48 h), suggesting that the principal role of Wnt-11 is to maintain NED and that the inability to do this leads to apoptosis. This would also be consistent with the known link between NED and resistance to apoptosis [22]. Wnt-11 is known to play a role in differentiation in several tissues, for example, promoting cardiomyogenic differentiation of mesodermal cells [23], mouse embryonic stem cells [24], endothelial circulating progenitor cells [25] and bone marrow mononuclear cells [9], and this occurs without effects on cell proliferation or survival in the latter two examples. Nevertheless, Wnt-11 could play an independent role in PCa cell survival and NED.

Wnt-11 inhibits AR transcriptional activity in LNCaP cells and NED is associated with a loss of AR expression [13]. Since ectopic expression of Wnt-11 in LNCaP cells reduced the level of AR (Figure 3d), it is possible that Wnt-11 induces NED indirectly by reducing AR expression. However, in contrast to what was observed in LNCaP-derived NE-like cell lines, which express no AR [13], expression of Wnt-11 did not lead to a complete loss of AR. Moreover, Wnt-11 and AR are co-expressed in other prostate cancer cell lines [2] and in prostate tumour cells (Figure 1i, j). Wnt-11 induction of NED was inhibited by four different inhibitors of PKA, suggesting that Wnt-11 activates activate PKA to induce NED. The signalling events that take place downstream of Wnt-11 during NED are not known, but they may involve activation of CREB, as observed during heart morphogenesis [10], since CREB is activated upon induction of NED and constitutively-active CREB can itself induce NED [26].

The role of Wnt-11 in NED appears to be restricted to PCa cells since ectopic expression of Wnt-11 did not increase expression of *NSE *in non-tumorigenic RWPE-1 cells, even though these cells have the potential to express this gene [18]. NE cells in the human prostate are thought to originate from a stem/progenitor cell that also gives rise to basal and secretory epithelial cells [27]. They can be distinguished from most NE-like cells derived from PCa cells because they are terminally differentiated and do not co-express luminal epithelial cell markers such as AR [13]. A recent study provided evidence that cells initiating PCa are derived from a malignantly transformed 'intermediate' cell that acquires stem cell-like activity, rather than from a transformed normal prostate stem cell [28]. If this holds true, Wnt-11 might specifically promote NED of PCa cells but not of normal progenitors, which could be important when considering Wnt-11 as a potential target for therapy.

A role for Wnt-11 in cell migration was first suggested by studies in Xenopus and zebrafish, where Wnt-11 promotes convergent extension movements during gastrulation [19,20]. More recently, Wnt-11 was shown to promote migration of intestinal epithelial cells [6]. Our demonstration that Wnt-11 is required for migration of PC3 cells and promotes invasion of LNCaP cells (Figure 5) underscores the potential of this molecule to promote PCa metastasis. Consistent with this possibility, Wnt-11 was detected in PCa cells in the process of invading the neural sheath (Figure 1d) and in PCa bone metastases (Figure 1g).

The level of Wnt-11 expression was higher in PCa compared to benign prostate but correlated inversely with Gleason grade. This was unexpected given the high level of expression of Wnt-11 in some high-grade tumours (Figure 1f) as well as in all prostate tumour bone metastases examined (Figure 1g and Table 1). Wnt-11 may play a role in metastasis independent of the state of differentiation or have different functions in low grade and high-grade PCa. For example, in low-grade PCa Wnt-11 might prevent apoptosis, whereas in high-grade PCa it could promote invasion and, through its effects on NED, promote progression to androgen-independence. The relative importance of these roles at each cancer stage and in each patient would likely depend on other mutations present in the tumour. Interestingly, there was a positive correlation between Wnt-11 expression and patient PSA levels above 10 ng/ml. This might seem at odds with the fact that Wnt-11 reduces AR activity in LNCaP cells. However, we previously showed that Wnt-11 does not inhibit AR activity in androgen-independent AR-positive prostate cancer cell lines [2]. Therefore it is possible that the Wnt-11-positive tumours from patients with high PSA levels contain an androgen-independent population of tumour cells. A more detailed analysis using a larger cohort of patients with follow-on data will be required to test these possibilities.

## Conclusions

This study shows that Wnt-11 protein levels are elevated in a significant proportion of human prostate tumours. In addition, it demonstrates that Wnt-11 is both necessary and sufficient for induction of neuroendocrine-like differentiation in prostate cancer cells and that this requires PKA activity. Finally, it shows that Wnt-11 is required for prostate cancer cell survival and promotes prostate cancer cell invasion. Taken together, these observations suggest that Wnt-11 is a potential therapeutic target in the treatment of prostate cancer.

## Methods

### Cell culture

LNCaP, PC3 and RWPE-1 cells were from the American Type Culture Collection (Rockville, MD, USA), purchased through LGC Promochem. Cell stocks were frozen within six months of receipt and, upon thawing, used for up to six months. LNCaP and PC3 cells were grown at 37°C, 5% CO_2 _in RPMI-1640 (Invitrogen) with 10% foetal bovine serum (FBS; First Link, UK) and antibiotics (100 units/mL penicillin, 100 μg/mL streptomycin, Invitrogen, UK). RWPE-1 cells were grown in Keratinocyte serum-free medium (KSFM) supplemented with bovine pituitary extract and EGF (Invitrogen, UK). LNCaP shRNA clones were grown in RPMI with 10% tetracycline-free FBS (Clontech, Takara Bio USA). For hormone-depletion experiments, LNCaP cells were cultured in phenol red-free RPMI containing 5% charcoal-stripped serum (CSS, First Link, UK). For transfections, cells were cultured in Opti-MEM (Invitrogen). The following PKA inhibitors were used: H89 (10 μM, Calbiochem, UK), KT5720 (5 μM, Calbiochem, UK), Rp-cAMPs (100 μM, Enzo Life Sciences, UK) and PKI (10 μM, Calbiochem, UK). Cells were incubated with inhibitors for 24 h.

### Plasmids, oligonucleotides and RT-PCR

A cDNA encoding human Wnt-11 (GeneCopoeia, Frederick, MD) was subcloned into pcDNA3.1 (Invitrogen) using EcoRI and XhoI. Plasmids expressing control and WNT11 shRNAs were generated by ligation of annealed oligonucleotides into pTER (21) with 5' - 3' sequences as follows:

Control: GATCCCCTTCTCCGAACGTGTCACGTTTCAAGAGAACGTGACACGTTCGG AGAATTTTTGGAAA

WNT11: GATCCCCGGACTCGGAACTCGTCTATTTCAAGAGAATAGACGAGTTCCG AGTCCTTTTTGGAAA

For RT-PCR, total RNA was extracted and analysed according to manufacturer's instructions (One step RT-PCR, Qiagen, UK). The primer sequences (5' - 3') were:

*WNT11 *- CGATGCTCCTATGAAGGTGAAA, CTTCCGTTGGATGTCTTGTTG

*NSE *- CTCATCAGCTCAGGTCTCTC, CCTTACACACGGCCAGAGAC

*GAPDH *- TGTTGCCATCAATGACCCCTT, CTCCACGACGTACTCAGCG

The primers for *ASCL1 *were as previously described [17]. The number of cycles used (32 for *WNT11 *and *NSE*, 40 for *ASCL1 *and 22 for *GAPDH*) was determined empirically so that there was a linear relationship between target quantity and product yield to permit semi-quantitative analysis. Analysis by real-time q-PCR was done using iTaq SYBR Green premix (BIO-RAD) and the following conditions: 95°C for 10 min, 40 cycles at 95°C for 15 s, 60°C for 1 min and a dissociation stage (95°C for 15 s, 60°C for 1 min, 95°C for 15 s, 60°C for 15 sec). *GAPDH *and *ASCL1 *primers used were as previously described [17]. Relative levels of mRNA expression were calculated using the Comparative CT Method, 2^-ΔΔCT ^(ΔΔCT: ΔCT sample - ΔCT calibrator) [29]. Experiments were performed in triplicate and statistical significance was analysed using Student's t-Test, comparing ΔCT (CT target - CT housekeeping) obtained from the calibrator.

### Transfections

For Wnt-11 overexpression, LNCaP and RWPE-1 cells were transiently transfected with pcDNA Wnt-11, empty vector or pEGFP-C1 (Clontech) using Fugene-HD (Roche) and assayed 48 h later. PKA inhibitors were added 24 hours after transfection. To generate LNCaP cell lines expressing control and WNT11 shRNAs, LNCaP/TR2 cells [30] were transfected with pTER control shRNA or WNT11 shRNA plasmids using Tfx-50 (Promega), selected in medium containing 6 μg/ml blasticidin and 300 μg/ml Zeocin (Invitrogen, UK) and colonies then picked and expanded. WNT11 expression was determined by RT-PCR analysis of clones cultured for 3 days in androgen-depleted medium. Three WNT11 shRNA clones with reduced expression of Wnt-11 and three control shRNA clones were selected and used for experiments. Experiments were conducted in medium containing 5% CSS and doxycycline to induce expression of the shRNA. Despite the use of a Tet-inducible LNCaP cell line and the pTER vector, expression of WNT11 shRNA in the expanded clones was only weakly affected by doxycycline (unpublished observations). siRNA transfections were performed according to the manufacturer's protocols using DharmaFECT 2 (Dharmacon, USA). PC3 cells were seeded in 6-well plates for 48 h before transfection using ON-TARGETplus SMARTpool WNT11 siRNA or ON-TARGETplus non-targeting pool (Dharmacon, USA). Transfected cells were processed for RNA extraction (after 24 and 48 h) or caspase activity (after 24, 48 and 72 h) or used in cell migration assays (72 h).

### Antibodies, western blotting and immunostaining

The following antibodies were used: mouse anti-NSE (BBS/NC/V1-H14, Dako), rabbit anti-cleaved caspase-9 (Asp330; 9501) and rabbit anti-cleaved PARP (Asp214; 9541) (both from Cell Signalling Technology), rabbit anti-AR (N20; sc-816, Santa Cruz Biotechnology), mouse anti-HSP60 (SPA-806, Stressgen) and goat anti-Wnt-11 (R&D systems, AF2647). Rabbit anti-Wnt-11 (H-95; sc-50360, Santa Cruz Biotechnology) was also used for immunohistochemistry to confirm the results using goat anti-Wnt-11. For immunocytochemistry, transfected RWPE-1 and LNCaP cells were fixed in 4% paraformaldehyde for 15 min, blocked with 5% BSA/PBS for 30 min and incubated with rabbit anti-Wnt-11 (1:20) and/or mouse anti-AR (clone AR441, Dako, 1:40) overnight at 4°C. Alexa Fluor^® ^488 goat anti-mouse IgG (H+L) and/or Alexa Fluor^® ^555 goat anti-rabbit IgG (H+L) (Invitrogen) were used as secondary antibodies (1:500, 60 min at room temperature) and nuclei were counterstained using 1.0 μM TO-PRO-3 (Invitrogen). Images were acquired on a LSM 510 laser scanning confocal microscope (Carl Zeiss). Preparation of extracts and western blotting were done as previously described (23). Blots were stripped and re-probed for HSP60 to control for gel loading. Human Prostate cancer and adjacent normal prostate tissues (tissue arrays and single tissue slides) were purchased from SuperBioChips Laboratories (single slides and TMA-CA3), Stretton Scientific (TMA-A222) and US BioMax Inc. (TMA-PR951). Samples were provided as formalin-fixed, paraffin-embedded and sectioned for histological analysis. Representative haematoxylin-eosin-stained sections were examined to evaluate the histopathological characteristics of the lesion to be analysed. The suppliers provided patient pathology data, when available. All samples were independently analysed for Gleason grade by a pathologist (MMW). Sections were deparaffinised and rehydrated using graded alcohol concentrations. Antigen retrieval was performed by incubation in 10 mM sodium citrate pH 6.0 and heating in a microwave oven at 560 W for 8 min. Endogenous peroxidase was quenched using 3% H_2_O_2 _for 30 min. Following blocking (30 min in PBS with 1.5% horse serum), sections were incubated with goat anti-Wnt-11 at 1:200, rabbit anti-Wnt-11 at 1:50 or anti-AR at 1:200 for 1 h at room temperature. Washing and antibody visualisation was done using the Vectastain Elite ABC Standard kit according to manufacturer's instructions (Vector Labs). TMA analysis was performed using an image analyser (ChromaVision ACIS II, Zeiss, Germany). Tissue samples were scored by MMW for Wnt-11 levels in prostate cancer epithelial cells (0 = no signal; 1 = positive, and 2 = strongly positive).

### Assays for morphology, apoptosis, cell number, migration and invasion

For morphological analysis, images of live cells were taken and analysed using ImageJ software http://rsb.info.nih.gov/ij. The numbers of cells with and without neurites longer than two cell bodies were counted in photomicrographs of control and WNT11 shRNA cells that had been cultured for 4 days in hormone-depleted medium. The data presented are the means derived from counting three fields of 100 cells from a representative experiment (n = 2). For caspase assays, trypsinised cells were plated at a density of 5000 cells per well in opaque 96-well cell culture plates (Perkin Elmer). At the indicated times after plating, an equal volume of Caspase-Glo 3/7 reagent (Promega) was added, the plate was incubated for 30 min and readings were taken using a luminometer. To normalise for cell number, MTT assays were conducted in parallel using the same number of cells plated in normal 96-well tissue culture plates. The number of viable cells was measured by adding 200 μl sterile MTT (3- [4,5-dimethylthiazol-2-yl]-2,5-diphenyltetrazolium bromide, 5 mg/ml) diluted in fresh medium to each well, loosely wrapping the plate in foil and incubating at 37°C for 3 h. The media were then replaced with 250 μl DMSO, followed by 25 μl glycine buffer (0.1 M glycine, 0.1 M NaCl, pH 10.5) and absorbance at 570 nm was read within 10 min. Details of PC3 cell migration assays were as described previously (24). Briefly, cells were trypsinised and plated at 2 × 10^4 ^cells/well onto 12-micron pore Transwell filters with a polycarbonate membrane (Corning). Following 6 h incubation, the MTT assays were used to determine the number of migrated cells and the results were plotted as Migration Index (Mi), which is the percentage of migrated cells compared to the total number of cells seeded. MTT assays were used to confirm that that there was no effect on total cell number during the 6 h time course of the experiments. LNCaP invasion assays were done in similar manner to Vias et al. [21], except that LNCaP cells were first transfected with vector or Wnt-11 plasmid and, after 48 hours, 5 × 10^5 ^transfected cells were plated on Matrigel-coated Transwell filters (BD Biosciences) in growth medium containing 1% FCS or 1% CSS supplemented with DHT to prevent expression of endogenous Wnt-11 (both conditions gave similar results). After a further 42 h, the number of invaded cells was determined by MTT assay. The total number of cells plated was also determined by MTT assay.

### Data analysis

All data were analysed as means ± standard deviation. Statistical significance was determined using Student's t-test or chi-squared test, as appropriate; results were considered significant for p < 0.05.

## Competing interests

The authors declare that they have no competing interests.

## Authors' contributions

PUO participated in the design of the study, acquired the majority of the data, analysed and interpreted data and drafted the manuscript; YK and MC acquired, analysed and interpreted data; MMW analysed and interpreted immunohistochemistry data and helped draft the manuscript; SD and RSD participated in the interpretation of data; JW participated in the study design and helped draft the manuscript; RMK conceived, designed and coordinated the study, acquired, analysed and interpreted data and drafted the manuscript. All authors read and approved the final manuscript.

## Author's information

SD's present address is UCL Cancer Institute, University College London.

Correspondence for RM should be sent to r.kypta@ic.ac.uk or rkypta@cicbiogune.es.

## Supplementary Material

Additional file 1 **q-PCR analysis of ASCL1 expression**. Expression of ASCL1 (relative to GAPDH) in LNCaP cells transfected with vector (Control) or Wnt-11 expression plasmid or cultured in hormone-depleted medium (CSS). Values are means +/- SD from three independent experiments, normalised to control. ASCL1 expression was significantly higher in Wnt-11-transfected cells than in control-transfected cells (p = 0.04). Click here for file

Additional file 2 **Comparison of transfection efficiency of LNCaP and RWPE-1 cells**. (a, b) Images of LNCaP (a) and RWPE-1 (b) cells transfected for 48 h with GFP plasmid. GFP is shown in green and cell nuclei in blue. A similar proportion of cells (5%) expressed GFP in both cell lines. (c, d) LNCaP (c) and RWPE-1 (d) were transfected with Wnt-11 plasmid and, after 48 h, stained for Wnt-11 (red). Wnt-11 staining was of a similar intensity in both cell lines. Click here for file
