# Supplementary figures and images for: Wnt-11 promotes neuroendocrine-like differentiation, survival and migration of prostate cancer cells
Source: Mol Cancer. 2010 Mar 10;9:55. doi: 10.1186/1476-4598-9-55 (PMC2846888; doi:10.1186/1476-4598-9-55)

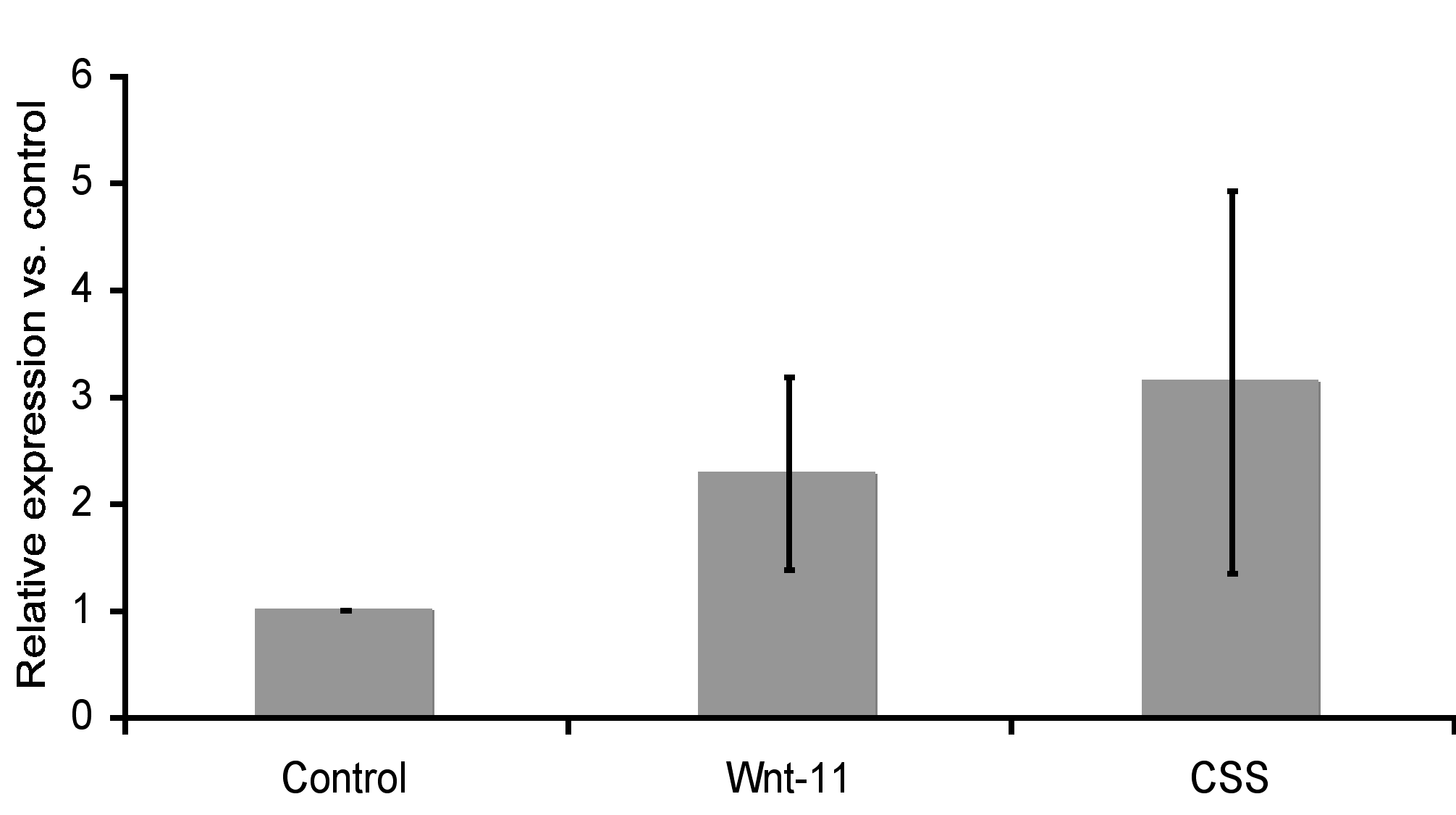

Supplement: Additional file 1 — q-PCR analysis of ASCL1 expression. Expression of ASCL1 (relative to GAPDH) in LNCaP cells transfected with vector (Control) or Wnt-11 expression plasmid or cultured in hormone-depleted medium (CSS). Values are means +/- SD from three independent experiments, normalised to control. ASCL1 expression was significantly higher in Wnt-11-transfected cells than in control-transfected cells (p = 0.04). [file 1476-4598-9-55-S1.TIFF]

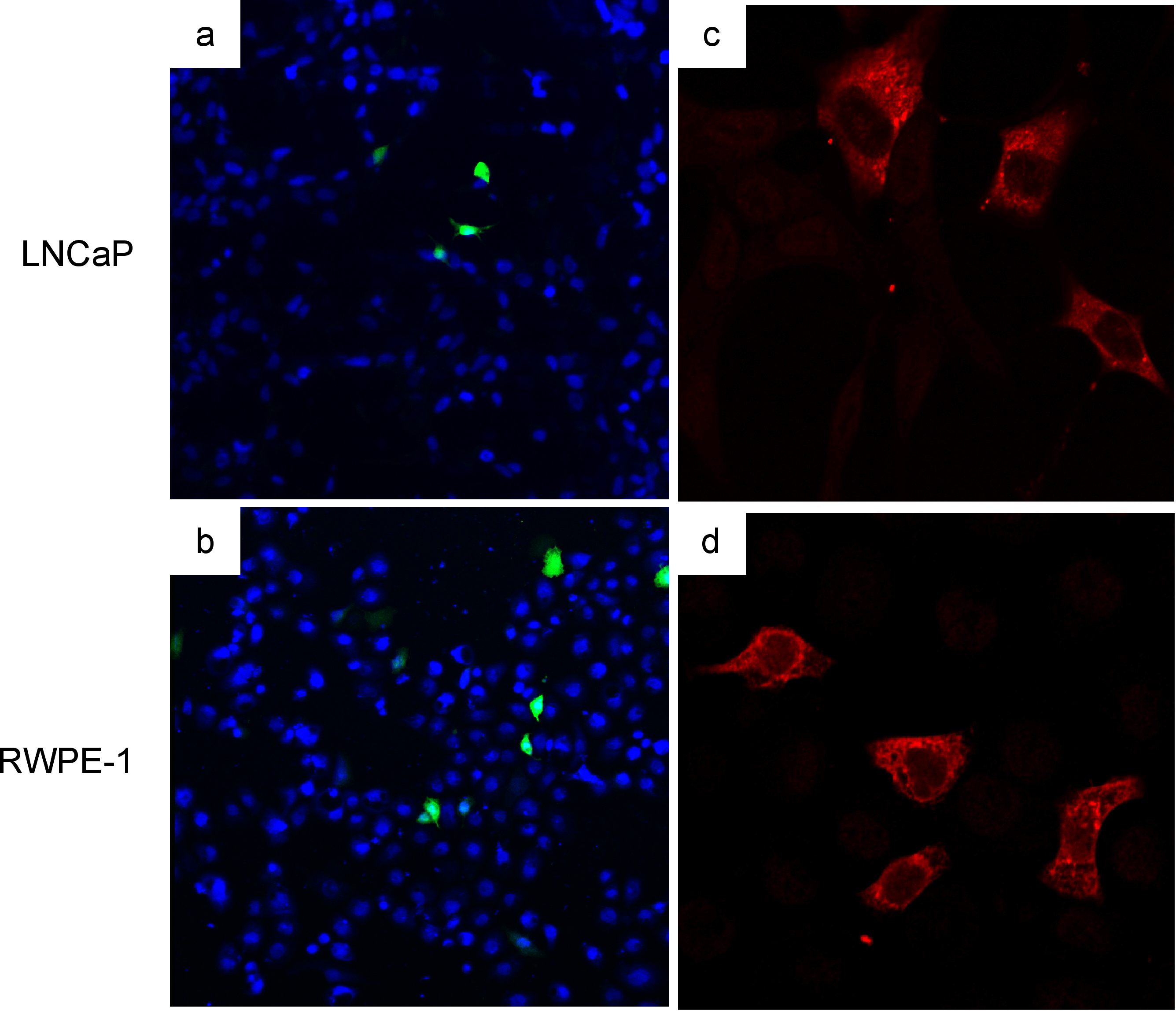

Supplement: Additional file 2 — Comparison of transfection efficiency of LNCaP and RWPE-1 cells. (a, b) Images of LNCaP (a) and RWPE-1 (b) cells transfected for 48 h with GFP plasmid. GFP is shown in green and cell nuclei in blue. A similar proportion of cells (5%) expressed GFP in both cell lines. (c, d) LNCaP (c) and RWPE-1 (d) were transfected with Wnt-11 plasmid and, after 48 h, stained for Wnt-11 (red). Wnt-11 staining was of a similar intensity in both cell lines. [file 1476-4598-9-55-S2.TIFF]
